# Effect of SGLT2 inhibitors on thiazolidinedione-induced changes in the volume status of patients with type 2 diabetes mellitus: a 6-month follow-up prospective study

**DOI:** 10.20945/2359-4292-2024-0485

**Published:** 2025-07-22

**Authors:** Yagmur Busra Unlusoy, Oguzhan Sıtkı Dizdar, Ali İhsan Gunal

**Affiliations:** 1 Department of Internal Medicine, Kayseri City Training and Research Hospital, Kayseri, Turkey; 2 Department of Internal Medicine and Clinical Nutrition, University of Health Sciences Kayseri City Training and Research Hospital, Kayseri, Turkey; 3 Department of Internal Medicine Division of Nephrology, Kayseri City Training and Research Hospital, Kayseri, Turkey

**Keywords:** Sodium-Glucose Transporter 2 Inhibitors, thiazolidinediones, water-electrolyte balance

## Abstract

**Objective:**

To ascertain the impact of combining sodium-glucose cotransporter 2 inhibitors (SGLT2is) with thiazolidinediones on fluid balance in patients with type 2 diabetes mellitus.

**Methods:**

This prospective study followed patients over a 6-month period, with data collected at three time points. The study commenced with the administration of pioglitazone on the same day. At the 3-month mark of the study, SGLT2is (dapagliflozin or empagliflozin) were subsequently integrated into the patients’ treatment regimens. At each time point, bioimpedance spectroscopy was employed to the volume status of the patients, and an assessment of their glycemic, renal, and lipid parameters was conducted. Their fluid status was evaluated on the basis of the overhydration value and the relative hydration index.

**Results:**

The study sample consisted of 60 type 2 diabetes mellitus patients with a mean age of 52.5 years. While notable increases in the mean overhydration value and relative hydration index were observed during the initial 3-month period (p < 0.001), a significant decline was evident in the second 3-month period (p < 0.001). However, no significant change in the adipose tissue index, fat mass, or body cell mass was found at the 6-month follow-up. Significant improvements were achieved in liver function test results, glycemic parameters, and the lipid profile. Renal parameters did not change significantly during the 6-months of follow-up.

**Conclusion:**

SGLT2is have been shown to be effective in improving fluid retention associated with thiazolidinediones and in maintaining euvolemic fluid status.

## INTRODUCTION

Thiazolidinediones (TZDs), a class of insulin-sensiti-zing drugs including pioglitazone, act as peroxisome proliferator-activated receptor subtype γ (PPAR-γ) activators. These drugs are effective and are increasingly used to treat patients with type 2 diabetes mellitus (DM) (^1^). Fluid retention represents the most common and serious side effect of TZDs, with an incidence ranging from 7% in patients receiving TZD monotherapy to 15% in those receiving combination therapy with insulin (^2^). Enhanced sodium and water reabsorption in the kidneys due to stimulation of PPAR-γ and the peripheral capillary leak phenomenon (^3^,^4^) have been proposed as mechanisms to explain the accumulation of peripheral edema; however, the most likely mechanism is increased renal sodium reabsorption and plasma volume expansion. Sodium-glucose cotransporter 2 inhibitors (SGLT2is) act by inhibiting the reabsorption of glucose from the kidney’s proximal tubule, resulting in glycosuria (^5^). SGLT2is exert mild natriuretic and glucosuria-induced osmotic diuretic effects, similar to those observed to occur from conventional diuretics (^6^). In addition, several randomized controlled trials have demonstrated that plasma volume decreased by 9.6% in individuals who have received the SGLT2i dapagliflozin for 24 weeks (^7^). The administration of SGLT2is has been demonstrated to exert a beneficial effect on the cardiovascular system and kidneys (^8^).

Fluid overload is a common occurrence among patients with type 2 DM, particularly in instances of concomitant heart failure and kidney disease (^9^). The degree of excess fluid accumulation can be accurately quantified using bioimpedance spectroscopy. A previous study indicated that total body water (TBW) levels tend to increase in individuals undergoing treatment with TZDs (^10^). Therefore, demonstrating by bioimpedance spectroscopy that the fluid overload caused by TZD treatment is reduced by SGLT2i treatment would inform the proper calibration of type 2 DM treatment. To date, no study has investigated whether TZD-induced hypervolemia can be alleviated with SGLT2is in patients with DM. A single study in mice showed that treatment with a combination of SGLT2i and pioglitazone can attenuate pioglitazone-induced fluid retention through osmotic diuresis (^3^), and a recent randomized trial revealed that pioglitazone-associated edema was reduced with the use of dapagliflozin (^11^). Nevertheless, no study has employed objective measurements of body water balance obtained via bioimpedance analysis (BIA). Therefore, the objective of this bioimpedance-based study was to ascertain the impact of combining SGLT2is with thiazolidinediones on fluid balance in patients with type 2 DM.

## METHODS

### Subjects and design

**Figure 1:**
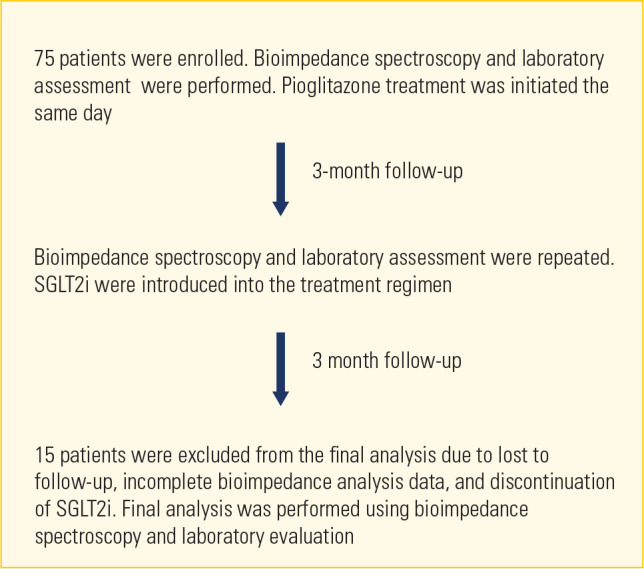
Flow of patients through the trial.

A total of 75 patients with type 2 DM were enrolled in a prospective, nonrandomized study at a tertiary care research hospital between December 2022 and June 2023. However, 15 patients were excluded from the final analysis for the following reasons: 10 were lost to follow-up, 2 had incomplete BIA data, and 3 discontinued SGLT2i treatment. The observation period of the study was 6 months. At the beginning of the study, bioimpedance spectroscopy was conducted to ascertain the baseline volume status of the patients (first measurement), and pioglitazone treatment was initiated the same day. These patients were not utilizing SGLT2is during the initial 3-month period of the study; they were solely receiving pioglitazone and other noninsulin antidiabetic medications, if any. After the completion of the third month of the study, a second bioimpedance spectroscopy measurement was taken, after which SGLT2is (dapagliflozin or empagliflozin) were introduced into the treatment regimens of the patients. SGLT2i were selected on a randomized basis and in accordance with the prevailing agents in our country. After the completion of the sixth month of the study, a third bioimpedance spectroscopy measurement was obtained to assess the final hydration status of the patients. In summary, the study visits were conducted at specific time points: baseline (the day when pioglitazone was first prescribed), 3 months (the day when the SGLT2i was first prescribed) and 6 months. A flow chart of the study is shown in **Figure 1.** Participants were excluded if they had a glomerular filtration rate of less than 30 mL/min/1.73 m^2^; a history of heart failure; malignant disease; type 1 DM; aspartate aminotransferase or alanine aminotransferase (ALT) levels greater than three times the upper limit of normal; insulin or diuretic treatment; or an active urinary tract infection. Therefore, the study excluded individuals taking major concomitant medications that had the potential to affect the volume status of patients. No concomitant medications were altered during the study period; therefore, no such alterations could have influenced the patients’ volume status.

SGLT2i: sodium-glucose cotransporter 2 inhibitors.

Blood and spot urine samples were collected from all participants at all study visits. Laboratory assessments included complete blood counts, routine biochemistry measurements, lipid panels (triglycerides, high-density lipoprotein [HDL] cholesterol, low-density lipoprotein cholesterol), and glycated hemoglobin (HbA1c) and spot urine protein-to-creatinine ratio measurements.

The study protocol was developed in accordance with the ethical principles set forth in the Declaration of Helsinki and was subsequently approved by the local ethics committee (approval number: 178639). All participants provided written informed consent prior to participation in this study.

### Bioimpedance spectroscopy method

At each study visit, body composition (including fluid status) was assessed using bioimpedance spectroscopy with a body composition monitor (BCM, Fresenius Medical Care, GmbH, Germany). The BCM was connected to four disposable electrodes, which were placed on the upper and lower limbs of the patients. Bioimpedance analysis was conducted in accordance with standard procedures, with measurements taken by a trained researcher with the patient in the supine position. For each patient, the following information was entered: sex, height (cm), body weight (kg), and arterial blood pressure (systolic and diastolic, mmHg). Noninvasive bioimpedance was employed to assess various indices related to the body’s fluid balance and tissue composition. These indices included hydration status, urea distribution volume, TBW, intracellular water (ICW), extracellular water (ECW), lean tissue index, fatty tissue index, fat mass and body cell mass. Extensive validation of the BCM has been conducted against all currently available gold standard methods in the general population (^12^). Fluid status was expressed as the overhydration value (OH). Patients were classified as negatively hydrated if their OH level was ≤ 0 L and as positively hydrated if their OH level was > 0 L. The relative hydration index, a marker of body fluid status, was calculated as OH/ECW × 100% (^13^).

### Statistical analysis

The data were analyzed with IBM Statistical Package for the Social Sciences (SPSS), version 29. To ascertain whether the data were normally distributed, the Kolmogorov-Smirnov and Shapiro-Wilk tests were employed. Mauchly’s test of sphericity was used to assess the homogeneity of the data distribution. The effects of the group and time main effects and their interactions on the parameters were analyzed using a repeated-measures of analysis of variance (Anova) for normally distributed data and the Friedman test for nonnormally distributed data. The Greenhouse-Geisser correction was applied to the parameters for which the assumption of sphericity, as postulated by Mauchly, was not met. In this instance, a valid correction was employed in the analysis of both parameters in which the homogeneous distribution, as determined by the Mauchly sphericity test, was accepted and parameters in which the assumption of sphericity was not met. To compare changes between measurements, the dependent-sample *t* test was employed for normally distributed parameters, whereas the Wilcoxon test was utilized for nonnormally distributed parameters. The results of the analysis are presented as the mean ± standard deviation and median (minimum-maximum) for quantitative data. The level of statistical significance was set at p < 0.05.

## RESULTS

The present study included a total of sixty patients with type 2 DM who commenced medication treatment with pioglitazone. The mean age of the patients was 52.5 years (ranging from 26 to 70 years), and 31% (n = 52) of the patients were male. After the completion of the third month of the study, empagliflozin was introduced into the treatment regimens of 33 subjects, while dapagliflozin was added to the regimens of 27 subjects. Hyperlipidemia and hypertension were the most common comorbidities, affecting 28 and 14 patients, respectively. At the time of inclusion, the most common antidiabetic treatment used by patients was biguanide, which was being utilized by 13 patients (21.6%). One patient was being treated with the glucagon-like peptide-1 (GLP-1) receptor agonist exenatide.

### The period of treatment with pioglitazone only (baseline to 3 months)

A significant increase in the mean OH value and relative hydration index (OH/ECW) was observed at the third-month measurement compared with the baseline values (p < 0.001 for both) (**Table 1**). A mean increase in the ECW of 0.23 L was observed. However, the adipose tissue index, fat mass, and body cell mass did not change significantly during the first 3 months of follow-up (p = 0.950, p = 0.309, and p = 0.532, respectively). The addition of pioglitazone to the treatment regimen resulted in significant reductions in both plasma glucose levels (baseline: 138 mg/dL, third month: 122 mg/dL, p < 0.001) and HbA1c levels (baseline: 7.35, third month: 6.8, p < 0.001). Along with these improvements in metabolic parameters, renal parameters also improved. The statistical analysis of the renal parameters of the patients revealed a significant increase in the estimated glomerular filtration rate (eGFR) (p < 0.001) and a decrease in the creatinine level (p < 0.001) and spot urine protein-to-creatinine ratio (p = 0.043) in the third month. Furthermore, analysis of the patients’ lipid and liver panels revealed decreases in the low-density lipoprotein (LDL) cholesterol, triglyceride, and ALT levels and an increase in the HDL cholesterol.

**Table 1:** Comparison of baseline, 3-month and 6-month bioimpedance measurements and laboratory results

	Baseline	3rd month	6th month	p-value between baseline and 3rd month	p-value between third and 6th months
Overhydration value, L	0.115 ± 1.15	0.832 ± 1.1	-0.225 ± 1.237	< 0.001	< 0.001
Relative hydration index	-0.25 (-21.4-12.2)	3.5 (-10.8-14)	-1.3 (-17.4-14.4)	< 0.001	< 0.001
Body mass index, kg/m^2^	32.4 ± 5.6	32.3 ± 5.7	32.3 ± 5.8	0.493	0.971
Fat mass, kg	46.8 (18.8-85.1)	46.2 (25.9-76.6)	46.3 (16.8-76.6)	0.950	0.464
Fat tissue index, kg/m^2^	16.8 (3.7-3.1)	16.6 (7.5-36.8)	17.2 (6.4-31)	0.309	0.548
Body cell mass, kg	22.1 (9.8-37.8)	20.7 (12.7-42.2)	21.5 (9.9-43.3)	0.532	0.726
Systolic blood pressure, mmHg	122.28 ± 15.87	120.41 ± 16.93	123.06 ± 14.73	0.453	0.234
Diastolic blood pressure, mmHg	74.75 ± 11.7	75.53 ± 10.91	77.13 ± 9.04	0.664	0.355
Blood urea nitrogen, mg/dL	12.5 (7-33)	13(7-25)	12 (9-26)	0.607	0.277
Creatinin, mg/dL	0.747 ± 0.162	0.745 ± 0.164	0.759 ± 0.165	< 0.001	< 0.001
eGFR, mL/dk/1,73/m^2^	101.32 ± 10.91	102.62 ± 9.75	100.32 ± 9.97	< 0,001	< 0.001
Sodium, mmol/L	139.383 ± 2.108	139.5 ± 2.318	139.65 ± 2.96	0.722	0.662
Potassium, mmol/L	4.547 ± 0.454	4.603 ± 0.446	4.54 ± 0.35	0.273	0.243
Spot urine protein-to-creatinine ratio, mg/g	75.15 (0-594.5)	74.3 (0-818.5)	75.845 (23-934.4)	0.043	0.394
Aspartate aminotransferase, U/L	19 (11-129)	18 (12-231)	19 (9-53)	0.055	0.454
Alanin aminotransferase, U/L	23 (5-147)	20.5 (9-178)	17 (8-59)	0.001	0.002
Fasting plasma glucose, mg/dL	138 (80-372)	122 (78-297)	116.5 (68-366)	< 0.001	0.334
HbA1C, %	7.35 (6-12.8)	6.8 (5.4-9.3)	6.45 (5.2-9.1)	< 0.001	< 0.001
Albumin, g/L	4.31 ± 0.36	4.34 ± 0.34	4.38 ± 0.348	0.451	0.457
LDL cholesterol, mg/dL	136 ± 37.6	123.4 ± 31.1	125.333 ± 29.745	0.023	0.609
HDL cholesterol, mg/dL	41 (23-61)	41 (26-86)	45 (23-75)	0.005	0.012
Triglyceride, mg/dL	193.5 (59-972)	191 (59-611)	169.5 (78-852)	0.015	0.317
White blood cell count, 10^3^/ µL	7.98 ± 2.02	7.69 ± 2.23	7.924 ± 2.288	0.152	0.363
Hemoglobin, g/dL	14,7 (10.6-18.6)	14,55 (10.8-13.6)	14.65 (9.2-18.1)	< 0.001	0.076
Platelet count, 10^3^/ µL	279.35 ± 54.4	281.5 ± 54.5	291.433 ± 70.955	0.669	0.207

Results expressed as the mean ± standard deviation and median (minimum-maximum).

eGFR: estimated glomerular filtration rate; LDL: low-density lipoprotein; HDL: high-density lipoprotein.

### The period of treatment with pioglitazone plus SGLT2i (3 months to 6 months)

A significant decrease was observed in the mean OH value and relative hydration index at the 6-month follow-up in comparison with the values at the 3-month follow-up (p < 0.001 for both) (**Table 1**). A mean ECW reduction of 0.7 L was observed. However, the adipose tissue index, fat mass, and body cell mass did not significantly change during the second 3 months of follow-up (p = 0.548, p = 0.464, and p = 0.726, respectively). No significant discrepancy in the decrease in the OH value was observed between empagliflozin and dapagliflozin usage (p = 0.652). The addition of SGLT2is to the treatment regimen resulted in a significant reduction in HbA1c levels (third month: 6.6%, p < 0.001). A comparative analysis of the laboratory values of the patients revealed an increase in the creatinine value and a decrease in the eGFR value between the second and third measurements (p < 0.001 for both). The decline in ALT levels persisted throughout the second 3-month interval (p = 0.002).

### Comprehensive analysis of the entire period (baseline to 6 months)

**Figure 2:**
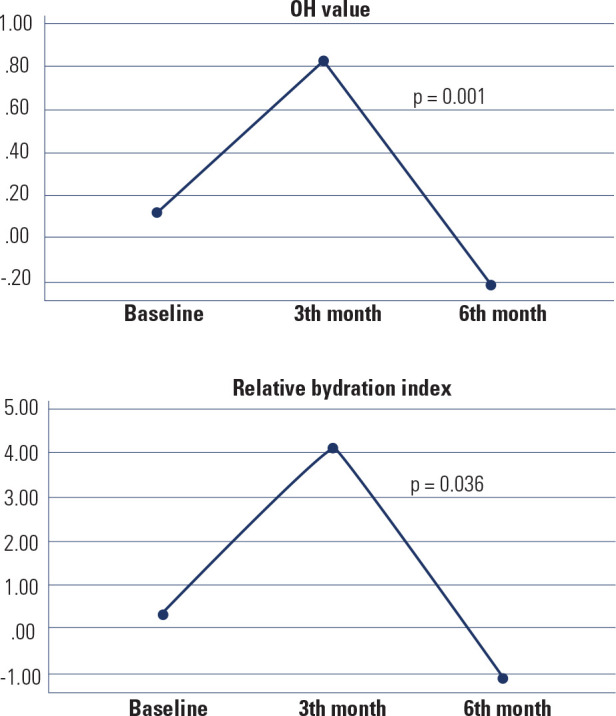
Changes in the overhydration value and relative hydration index from baseline at 6 months.

**Figure 3:**
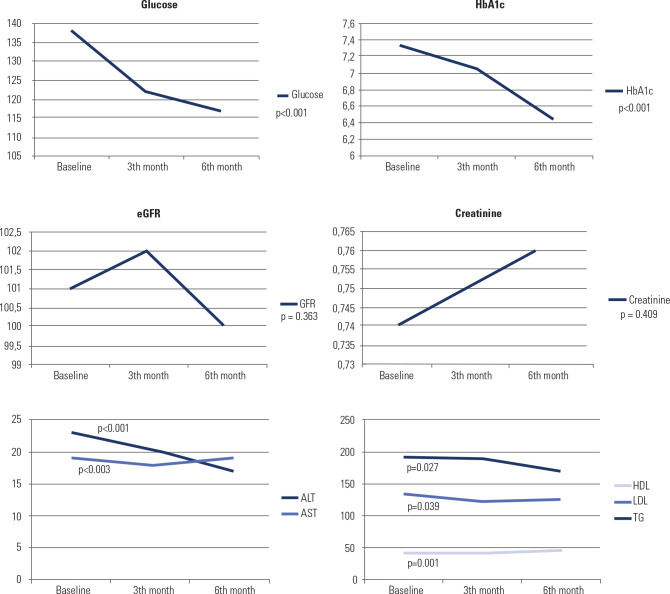
Changes in laboratory values from baseline at 6 months.

HbA1c: glycated hemoglobin; eGFR: estimated glomerular filtration rate; GFR: glomerular filtration rate; HDL: high-density lipoprotein; LDL: low-density lipoprotein.

The OH value and relative hydration index of the patients increased until the end of the third month, followed by a decrease in the subsequent 3 months. At the end of the sixth month, the net changes consisted of decreases in both the OH concentration and the relative hydration index (p = 0.001 and p = 0.036, respectively) (**Figure 2**). The adipose tissue index, fat mass, and body cell mass did not change significantly during the 6-month follow-up period (p = 0.242, p = 0.646, and p = 0.845, respectively). The changes in laboratory values from the baseline at six months are presented in **Figure 3**. Significant improvements were achieved in liver function tests, glycemic parameters and lipid profiles. Renal parameters did not change significantly during the 6 months of follow-up.

OH: overhydration.

## DISCUSSION

The present study yielded evidence concerning the ability of SGLT2is to prevent volume overload in patients with DM who are receiving pioglitazone therapy. Treatment with SGLT2is effectively decreases the ECW (mean 0.7 lt) after 3 months of treatment. Therefore, patients using pioglitazone are particularly likely to benefit from treatment with an SGLT2i. Furthermore, no evidence of volume depletion was observed, and no significant differences in renal parameters were identified in these patients when an SGLT2i was added to pioglitazone treatment compared with the baseline values. In this context, SGLT2is appear to exert a volume-controlling effect rather than a volume-depleting effect. Additionally, the application of bioimpedance spectroscopy, as employed in this study, provides an objective methodology and outcomes that can be readily integrated into routine clinical practice.

The OH level, as measured by bioimpedance spectroscopy, is a reliable indicator of fluid retention. This reflects the body’s fluid balance over the previous days and weeks. Extracellular volume expansion, represented by an increase in OH or the relative hydration index as measured by BIA, may contribute to an increased risk of disease in organs such as the heart and kidneys (^14^). Therefore, a promising cardiorenal protective mechanism in these patients may be the reduction in extracellular volume expansion caused by SGLT2 inhibition.

The results of this study are consistent with previously reported results regarding the occurrence of fluid retention in patients treated with pioglitazone (^15^). This finding aligns with the documented prevalence of edema, a common adverse event associated with TZDs. The clinical significance of these alterations in TBW associated with TZDs requires further investigation, particularly in view of the high prevalence of associated comorbid conditions, such as chronic kidney disease (CKD), heart failure, hypertension and obesity. Recent clinical studies in patients with type 2 DM with CKD or heart failure have demonstrated that SGLT2is reduce extracellular volume (^16^-^18^). Similarly, the present results indicate that SGLT2is, in contrast to pioglitazone, have the capacity to reduce fluid retention. Furthermore, combination therapy with both agents did not exacerbate fluid retention; rather, it mitigated the adverse effects, potentially through SGLT2i-induced osmotic diuresis. Therefore, the results of our study suggest that when pioglitazone is added to the treatment regimens of patients with these diseases, SGLT2is may mitigate the adverse effects of the pioglitazone-induced volume increase. Additionally, no notable discrepancy was observed in the volume-controlling effect between empagliflozin and dapagliflozin usage.

The results of one study indicated that SGLT2i produced greater electrolyte-free water clearance than other sodium-driven diuretics did (^19^). Therefore, osmotic diuresis resulting from SGLT2 inhibition leads to increased fluid clearance from the interstitial fluid space relative to the blood. This phenomenon has the potential to alleviate congestion while exerting minimal effects on blood volume and organ perfusion (^19^). This more pronounced modulation of interstitial fluid by SGLT2is may be related to its volume control effect rather than a volume-depleting effect, as demonstrated in this study.

The results of a recent study, which employed the combination of pioglitazone and SGLT2i, corroborate our findings (^11^). The observed prevalence of pioglitazone-induced peripheral edema in this study is relatively low in comparison with that reported in the earliest studies in which patients received pioglitazone monotherapy (^2^). However, since BIA was not performed in that study, the volume changes of the patients were not objectively demonstrated. Our study is the first to demonstrate that SGLT2is have a reducing effect on pioglitazone-related fluid retention, as evidenced by objective BIA measurements. Moreover, the results of the present study indicated that bioimpedance spectroscopy has considerable potential as a tool to improve the regulation of type 2 DM treatment regimens, as indicated by a previous study (^10^).

Although previous studies have indicated that SGLT2is may have a blood pressure-lowering effect (^20^), our study did not demonstrate this effect, which was not the primary focus of the study. Furthermore, an increase in hemoglobin levels following the initiation of an SGLT2i has been previously documented (^21^), which might also reflect changes in fluid status, with hemoglobin values becoming less diluted following a decrease in OH. However, in our study, the increase in hemoglobin after the initiation of SGLTi treatment did not reach statistical significance. Fluid retention is strongly associated with proteinuria in patients with CKD (^22^,^23^). However, our findings did not indicate a significant change in proteinuria levels with the reduction in fluid volume accompanying SGLT2i treatment.

Our results showed that both SGLT2is and pioglitazone were effective at improving glycemic and lipid parameters, which aligns with the findings reported in previous studies on patients with type 2 DM (^11^). However, combination treatment with SGLT2is and pioglitazone did not result in a reduction in fat tissue mass or body weight during the 6 months of follow-up, a finding that contrasts with some previous observations in obese patients with DM (^24^). A number of trials have also documented the beneficial impact of pioglitazone in patients with steatohepatitis, as well as its ability to reduce serum aminotransferase levels (^25^,^26^). Similarly, treatment with pioglitazone resulted in a notable reduction in serum ALT levels in our study.

Notably, the present study is subject to certain limitations. The study was conducted with a relatively small sample size, precluding the drawing of definitive conclusions. The study was not a randomized controlled trial, which introduces the possibility of bias or imprecision in the results. The long-term effects of SGLT2i on fluid status remain unclear, particularly in the context of extended use over periods exceeding 6 months.

In conclusion, TZDs may induce or intensify the development of fluid retention. SGLT2i treatment demonstrated efficacy in ameliorating TZD-induced fluid retention and maintaining euvolemic fluid status. It has also been demonstrated that the combination of TZDs with an SGLT2i results in synergistic and complementary improvements in glycemic parameters, lipid profiles, and liver function tests, with no adverse effects on renal parameters.
